# Surgical robot-assisted tripod percutaneous reconstruction technique combined with bone cement filling technique for the treatment of acetabular metastasis

**DOI:** 10.3389/fbioe.2023.1153394

**Published:** 2023-04-28

**Authors:** Zhen Huang, Kun-Peng Zhu, Jian-Ping Hu, Yu-Run Zhu, En-Jie Xu, Xiao-Long Ma, Yong-Jie Wang, Chun-Lin Zhang

**Affiliations:** Department of Orthopedic Surgery, Institute of Bone Tumor, Shanghai Tenth People’s Hospital Affiliated to Tongji University, Tongji University School of Medicine, Shanghai, China

**Keywords:** surgical robot, tripod, reconstruction, bone cement, acetabular metastasis

## Abstract

**Background:** Acetabular metastasis is a type of metastatic bone cancer, and it mainly metastasizes from cancers such as lung cancer, breast cancer, and renal carcinoma. Acetabular metastasis often causes severe pain, pathological fractures, and hypercalcemia which may seriously affect the quality of life of acetabular metastasis patients. Due to the characteristics of acetabular metastasis, there is no most suitable treatment to address it. Therefore, our study aimed to investigate a novel treatment technique to relieve these symptoms.

**Methods:** Our study explored a novel technique to reconstruct the stability of the acetabular structure. A surgical robot was used for accurate positioning and larger-bore cannulated screws were accurately inserted under the robot’s guidance. Then, the lesion was curetted and bone cement was injected through a screw channel to further strengthen the structure and kill tumor cells.

**Results:** A total of five acetabular metastasis patients received this novel treatment technique. The data relating to surgery were collected and analyzed. The results found that this novel technique can significantly reduce operation time, intraoperative bleeding, visual analogue score scores, Eastern Cooperative Oncology Group scores, and postoperative complications (e.g., infection, implant loosening, hip dislocation) after treatment. Follow-up time ranged from 3 months to 6 months, and the most recent follow-up results showed that all patients survived and no acetabular metastasis progressed in any of the patients after surgery.

**Conclusion:** Surgical robot-assisted tripod percutaneous reconstruction combined with the bone cement filling technique may be a novel and suitable treatment in acetabular metastasis patients. Our study may provide new insights into the treatment of acetabular metastasis.

## 1 Introduction

Acetabular metastasis mainly occurs in advance-stage tumors and can lead to severe pain, pathologic fractures, and functional limitation in patients (Yang et al., 2021; de l'Escalopier et al., 2022). Most acetabular metastasis patients take potent analgesics to relieve pain (Goetz et al., 2004). However, with analgesics drug adaptation, these potent analgesics may no longer alleviate the painful feelings of patients. The treatment of acetabular metastasis differs from that of primary bone tumors, and radiotherapy and chemotherapy are mainly used to control the progression of acetabular metastasis tumors (Felden et al., 2015; Giordano et al., 2018). However, pure chemotherapy and radiotherapy cannot alleviate the pain caused by acetabular metastasis. Therefore, surgical intervention may be a potential treatment for acetabular metastasis. Currently, the Harrington surgical staging method (I, metastasis accumulated locally on the articular surface; II, metastasis involved in the medial wall of the acetabulum; III, the inner wall, top, and edge of the acetabulum destroyed by metastasis; IV, isolated acetabular metastasis) is used to stage acetabular metastasis (Harrington, 1981; Nayar et al., 2022). Different surgical methods are selected depending on the different Harrington stages of acetabular metastasis. Traditional open surgical methods require wide-range demolition and construction, which can lead to multiple intraoperative and postoperative complications (Felden et al., 2015). Hence, the traditional open surgical methods may not be suitable treatments for all acetabular metastasis patients.

Therefore, minimally invasive treatment is needed for acetabular metastasis. The percutaneous tripod reconstruction technique for the minimally invasive treatment of acetabular metastases was first reported by Yang et al. (2020) in 2020. They formed tripod structures via the percutaneous placement of three large-bore cannulated screws under fluoroscopy to strengthen the mechanical axis of the acetabulum, increase stability, and reduce pain. However, there may be shortcomings to this reconstruction method: the position and depth of the screws cannot be accurately grasped under percutaneous fluoroscopy, the bone structures are significantly destroyed in Harrington stage II and III acetabular metastasis, and tripod fixation may not prevent the progression of bone structure destruction. Therefore, our present study aimed to investigate a novel integrity technique for treating acetabular metastasis.

## 2 Materials and methods

Patients with symptomatic metastasis (severe pain and activity disorder) in the acetabulum were selected in our present study. Patients with obviously disrupted hip articular surface or protrusion and ipsilateral femoral heads were excluded. A total of five acetabular metastasis patients who received treatment in Shanghai Tenth People’s Hospital of Tongji University from 1 July 2022 to 31 November 2022 participated in our study. We have designed novel, comprehensive, minimally invasive techniques for treating acetabular metastasis patients. With the help of a TINAVI robot (TINAVI Medical Technologies, China), we used the anterior column screw (anterior superior iliac spine to pubic symphysis), posterior column screw (ischial tuberosity to the sacroiliac joint), and trans-columnar screw (anterior inferior iliac spine to posterior superior iliac spine) combined with bone cement injection to stabilize the acetabular structure and relieve pain. All patients underwent pelvic X-ray, computed tomography (CT), and magnetic resonance (MR) imaging scans before surgery. Similarly, pelvic X-ray and CT imaging scans were undertaken after surgery to clarify the postoperative situation. Two experienced orthopedic oncologists independently defined the Harrington stage of the periacetabular lesions. All patients signed the informed consent before the surgical procedure. Our study conformed to the ethical guidelines of the Declaration of Helsinki and was approved by the Ethics Committee in our hospital.

The basic information of all patients, including age, gender, diagnosis, Harrington stage, American Society of Anesthesiologists (ASA) score, Eastern Cooperative Oncology Group (ECOG) score and visual analogue score (VAS) of pain, operative time, intraoperative bleeding, and complications, were recorded in detail before and after surgery. Tissue specimens determined the pathology diagnosis of patients after surgery and two independent pathologists evaluated the results.

## 3 Surgical technique

After the patient was under general anesthesia, the buttocks were raised, and the operation side abdominal perineum, pelvis, and operation side lower limb were exposed. The skin was protected by routine disinfection and napkins. After fixing the locator on the anterior superior iliac spine, the O-arm imaging machine was scanned for registration. After the image was input into the robot console, the robot evaluated the required length and width of the screw. The screw length from the anterior inferior iliac spine to the symphysis pubis was 6.5 mm*105 mm in patient 1, 7.3 mm*100 mm in patient 2, and 7.3 mm*100 mm in patient 3. The screw length from the anterior inferior iliac spine to the posterior superior iliac spine was 6.5 mm*105 mm in patient 1, 6.3 mm*90 mm in patient 2, 6.3 mm*110 mm in patient 3, 7.3 mm*105 mm in patient 4, and 6.5 mm*120 mm in patient 5. The length of the screw from the ischial tuberosity to the sacroiliac joint was 7.3 mm*100 mm in patient 2, 7.3 mm*115 mm in patient 3, 7.3 mm*105 mm in patient 4, and 6.5 mm*120 mm in patient 5. The anterior and posterior columns were not fixed by screws if there was no tumor erosion or severe bone destruction that meant it could not be fixed.

Meanwhile, the acetabular channel was designed from the front to the medical side of the acetabulum. After positioning the body surface, four anchor points were inserted into the guide needle, and the length and position of the guide needle were scanned again for certainty. Hollow drill to enlarge, three hollow screws to the remaining 20mm, and pull out the guide wire. After the acetabular roof channel expanded, a biopsy needle was used to implant the acetabular roof. A small amount of cortical bone and bone marrow was removed, and sent to examine the frozen section and paraffin section pathological examination. The bone cement was prepared and, after the lesion was scraped, an appropriate amount was injected into the top of the acetabulum using the hollow screw to fill the lesion site and kill tumor cells. Fluoroscopy showed that the bone cement filled the lesion site without extravasation. The cannulated screw was quickly screwed in and the position of the screw was scanned again and found to be consistent with the predetermined channel. The internal fixation position was good and no leakage of bone cement was observed. The minimally invasive incisions were sutured and covered with sterile dressings.

## 4 Statistical analysis

GraphPad Prism 8.3.0 software (GraphPad Software, United States) was used for statistical analysis in our study. Data were shown as mean - standard deviation. Student’s t-test was applied to the changes of parameters between preoperative and postoperative data, and a *p*-value less than 0.05 was considered statistically significant.

## 5 Results

A total of five acetabular metastasis patients were treated with our robot-assisted tripod percutaneous reconstruction technique combined with the bone cement filling technique, and their demographic and clinical characteristics were recorded in our study.

**FIGURE 1:**
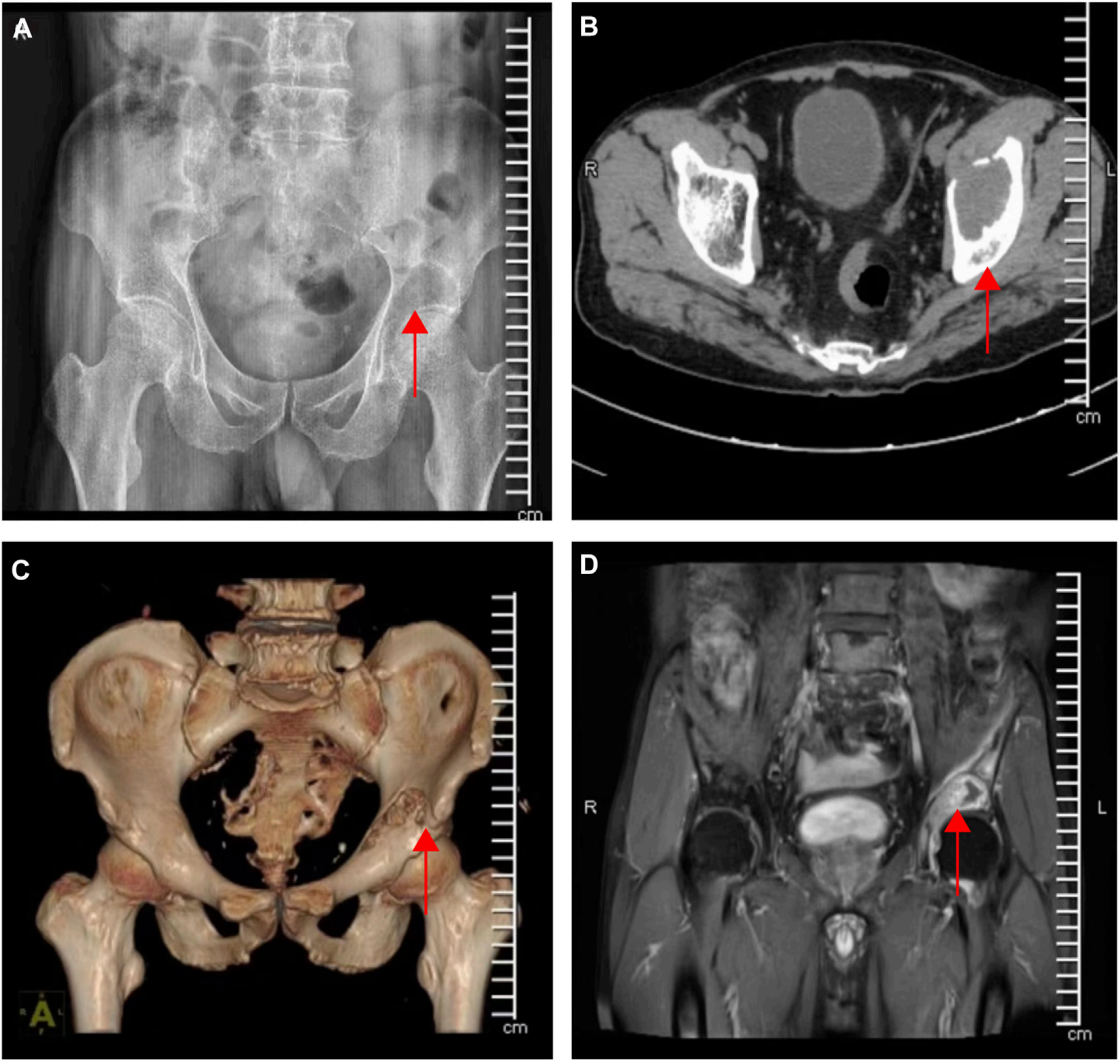
Preoperative imaging data of acetabular metastases. **(A)** Lesion under X-ray radiographs **(B–C)** CT and 3D reconstruction of the lesion **(D)** Magnetic resonance imaging of the lesion (the red arrow indicates the site of the lesion).

**FIGURE 2:**
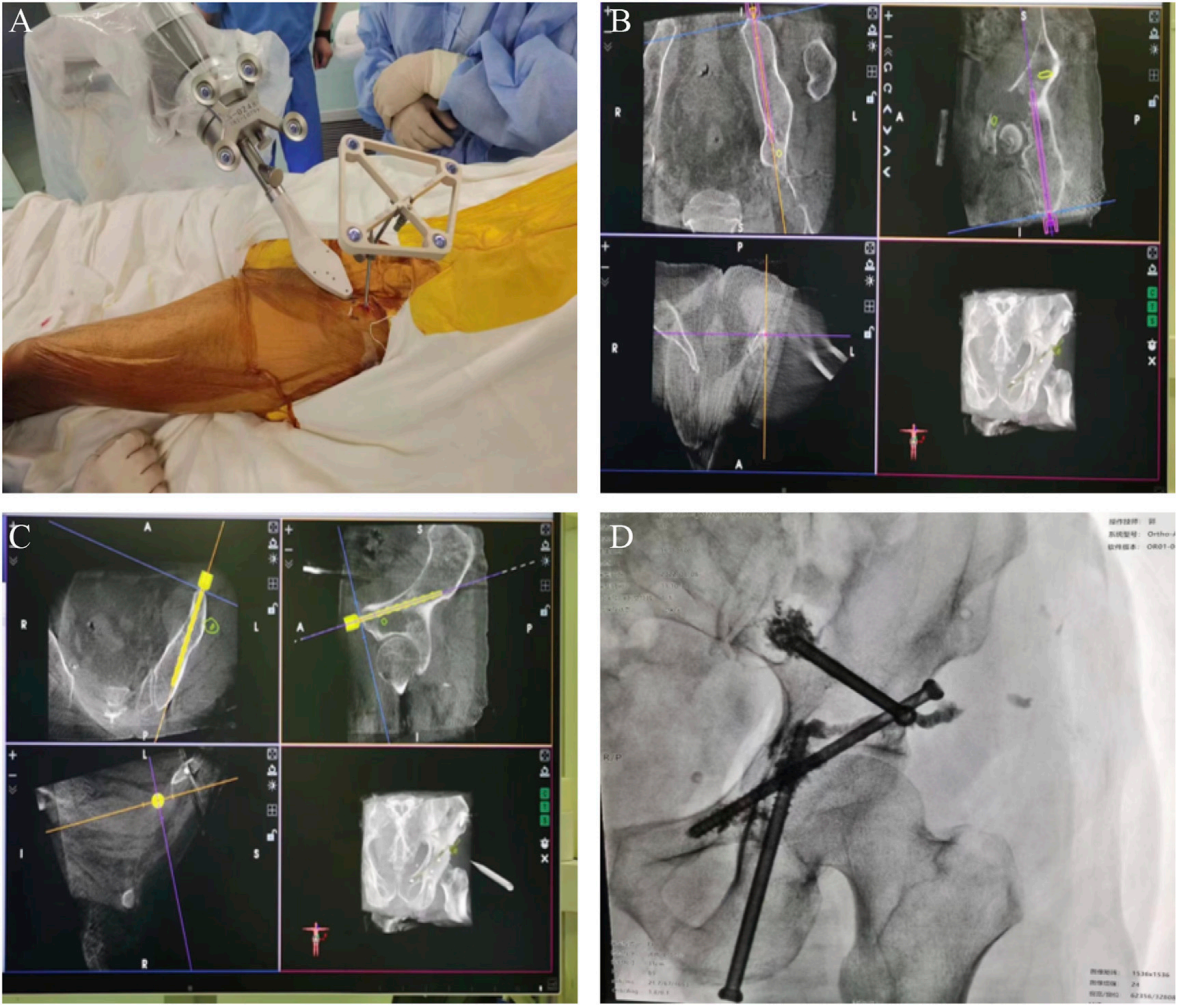
Intraoperative orthopedic robot for accurate positioning and imaging at the completion of surgery. **(A–C)** Accurately located screw channels with the help of orthopedic robot **(D)** X-ray radiographs of screw fixation and cement filling when the surgery was completed.

**FIGURE 3:**
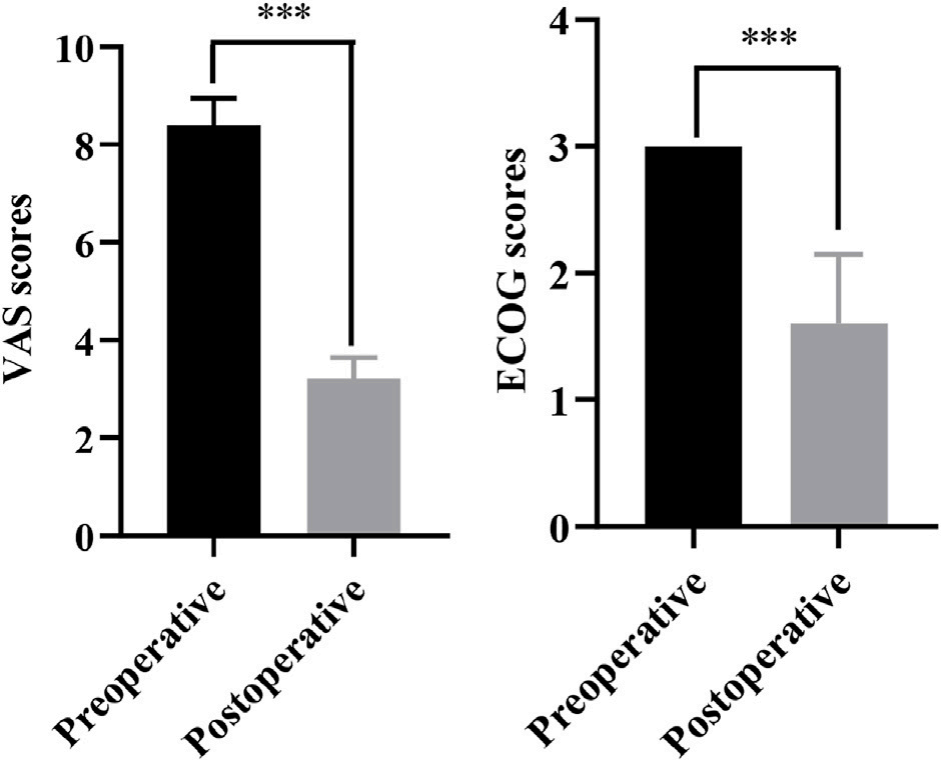
The comparison of VAS scores and ECOG scores between preoperative and postoperative. ***p < 0.001.

**FIGURE 4:**
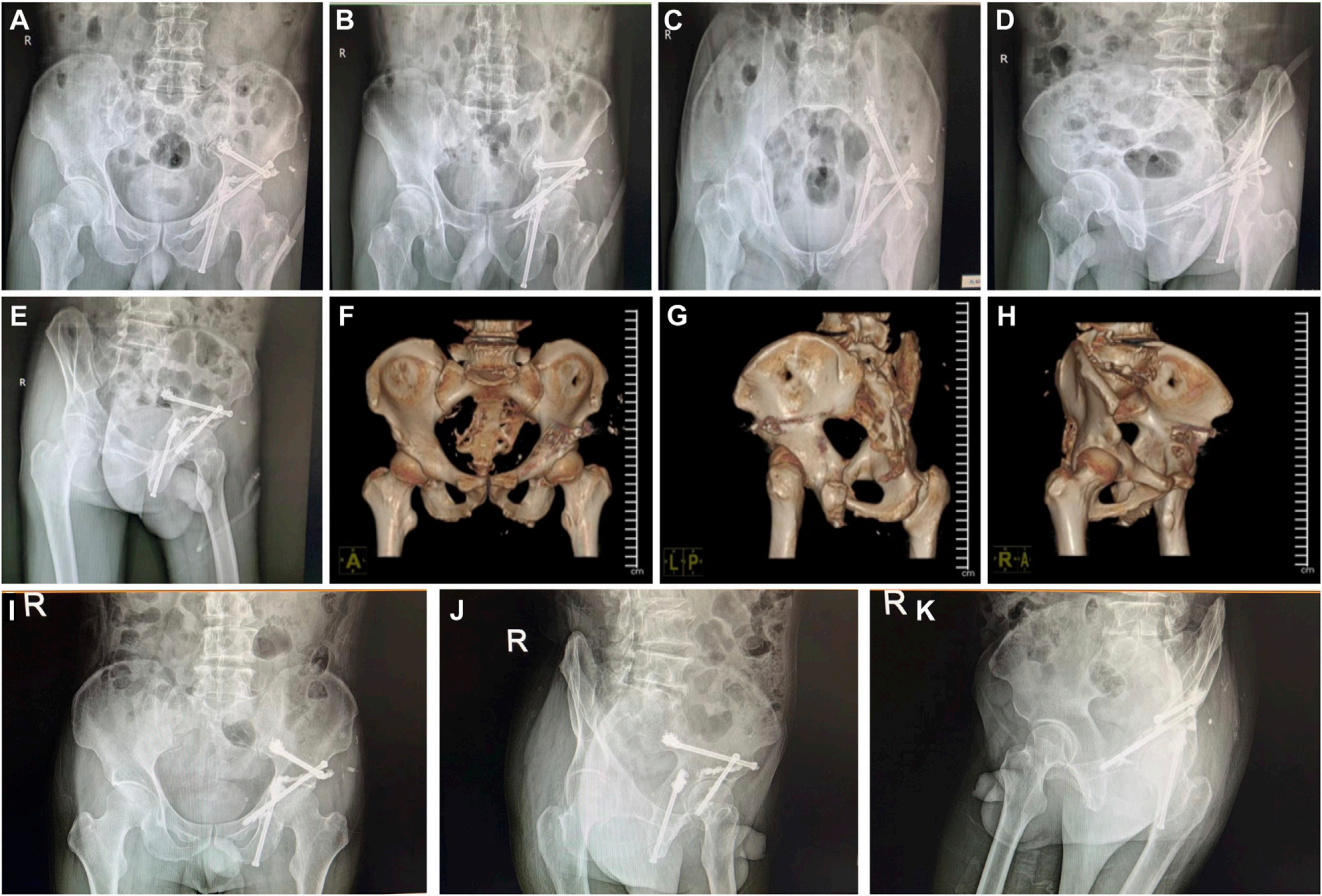
Postoperative review of X-ray radiographs and CT 3D reconstruction, and the postoperative follow-up X-ray images of the patient. **(A–E)** Different angles of X-ray radiographs on the pelvis to evaluate the operation **(F–H)** CT 3D reconstruction of the pelvis to assess the operation. **(I–K)** Different angles of X-ray radiographs on the pelvis to evaluate the operative area in the postoperative follow-up patient.

Our study had two male and three female patients and the average age was 73 years old. The pathological results of all patients were metastatic adenocarcinoma, and four patients presented with Harrington class-III lesions (Table 1). CT scans and three-dimensional reconstruction techniques were applied to specific acetabular metastasis (Figure 1). The scores of preoperative VAS pain, ECOG, and ASA were recorded in Table 2. None of the patients had received prior treatment for acetabular metastasis. With the help of the TINAVI robot, we only needed one time radiation before surgery to design the screw channels and one after the screws were in place to examine the locations, which minimized the radiation exposure for both patients and surgeons and accurately estimated the size of scores needed (Figure 2). Intraoperative treatment times for acetabular metastasis were 50 min in patient 1, 60 min in patient 2, 40 min in patient 3, 40 min in patient 4, and 35 min in patient 5. Patient 1 had an intraoperative hemorrhage of 300 mL and 4.5 cement injection; patient 2 had a 100 mL hemorrhage and 5.5 mL cement injection. The intraoperative hemorrhages for all patients were less than 50 mL and 4 mL cement injection. All patients were operated on within an hour and patients experienced significant pain relief in metastatic lesions, with the mean score of VAS pain being 3 or 4 on the first day after treatment and the ECOG score decreased to 1 or 2 in all patients after treatment (Table 2; Figure 3). On the second day after surgery, the patient underwent X-ray and CT 3D reconstruction to evaluate the operative site, which showed that lesions were treated sufficiently, all screws were in position, and there were no leakages of bone cement (Figures 4A–H). Patients proceeded to the next stage of treatment within 1 week after treatment. In the most recent follow-up (172 ± 42 days), all patients could walk independently or used walker assistance or a wheelchair to attend follow-up, implant loosening or failure was not observed, and none of the patients still had excruciating pain. All patients survived postoperatively and none of the patients underwent total hip arthroplasty due to the continuous progression of acetabular metastasis lesions. The follow-up imaging demonstrated the screws were in position and there was no progression in the metastasis lesions (Figures 4I–K).

**TABLE 1:** Clinical characteristics of the five patients.

Patient number	Age	Gender	Pathology diagnosis	Harrington stage	Other systemic diseases (e.g., hypertension, diabetes)
1	68	Female	Metastatic adenocarcinoma	II	Yes
2	74	Male	Metastatic adenocarcinoma	III	Yes
3	62	Male	Metastatic adenocarcinoma	III	Yes
4	87	Female	Metastatic adenocarcinoma	III	Yes
5	74	Male	Metastatic adenocarcinoma	III	Yes

**TABLE 2:** Clinical outcome of the five patients.

Patient number	VAS scores			ECOG scores			ASA scores	Surgical time (mins)	Cement (mL)	Intraoperative bleeding (mL)
	Preoperative	Postoperative	*p*-Value	Preoperative	Postoperative	*p*-Value				
1	8	3	<0.001	3	2	<0.001	3	50	4.5	50
2	8	3		3	2		3	60	5.5	30
3	8	4		3	1		3	40	4	40
4	9	3		3	1		3	40	4	50
5	9	3		3	2		3	35	4.5	40

## 6 Discussion

With the progression of targeted therapy, chemotherapy and radiotherapy technology, the overall survival times of malignant tumor patients have been continuously prolonged (Liu et al., 2022; Loibl et al., 2022; Spina et al., 2022). Therefore, the number of acetabular metastatic patients is gradually increasing. Traditional minimally invasive treatments, including pure bone cement injection, tumor lesion curettage combined with bone cement injection, and local microwave ablation combined with bone cement injection, are mainly used in Harrington stage I and in some of the acetabular metastatic patients with Harrington stage II (Lozano-Calderon et al., 2016; Wallace et al., 2016). However, for most acetabular metastatic patients, their Harrington stages are mostly III or IV, and present minimally invasive treatments are no longer applicable.

Most orthopedic surgeons may think open surgery is still suitable for acetabular metastasis. Meanwhile, most patients with acetabular metastasis are elderly and have concurrent multisystem diseases (Gao et al., 2020). The poor physical conditions of patients may result in open surgery being unfeasible. Even after the surgery is performed, the incidence of surgical complications is relatively higher than in young patients, as confirmed by studies that have shown that the main complications are pin migration and infection in acetabular metastasis patients who undergo open surgery (Houdek et al., 2020; Lavignac et al., 2020; Plaud et al., 2022). Therefore, open surgery may not be suitable for treating acetabular metastasis patients who are elderly and with multiple diseases. The percutaneous tripod reconstruction technique for minimally invasive treatment of acetabular metastasis was first reported by Yang et al. (2020), who found that percutaneous placement of three large-bore cannulated screws under fluoroscopy can increase acetabular stability, alleviate pain, and allow partial loading. Araneta et al. (2022) reported that minimally invasive procedures for patients with periacetabular metastasis might avoid the need for complex hip replacement and its associated postoperative complications. Moreover, the study also demonstrated that minimally invasive stabilization in the treatment of periacetabular metastasis could relieve pain, improve function, and allow rapid initiation of radiation and chemotherapy (English et al., 2021). In patients who suffered from Harrington stage III acetabular metastasis, their acetabular bone structures were destroyed and the bone destruction continued to progress. This condition was confirmed by Yang et al. (2020), who demonstrated that 25% of patients who received the percutaneous tripod reconstruction technique finally underwent total hip arthroplasty (THA) treatment with persistent pain or local disease progression.

Our study further developed tripod technology, combining an orthopedic surgical robot and bone cement filling technology, which may compensate for the shortcomings of tripod technology. The TINAVI robot is the third generation of orthopedic surgical robot developed in China. It can be used in various orthopedic surgeries, including spinal fractures, femur fractures, pelvis fractures, and others (Wu et al., 2019; Luengo-Matos et al., 2022). Orthopedic surgical robots can help the surgeon make precise surgical positionings, shorten operation time, and reduce the number of intraoperative X-rays and radiation doses (Jiang et al., 2014; Guo et al., 2022). Without the help of the TINAVI robot, surgeons spend more time locating the surgical site, multiple rounds of radiation are required, and most parts of the final positioning are not as precise as when using the TINAVI robot. When comparing the clinical outcomes with the other surgical methods in the treatment of acetabular metastasis reported by Yang et al. (2020), Lavignac et al. (2020), Houdek et al. (2020), and Plaud et al. (2022), our treatment has the shortest surgical time (45 ± 10) and has minimal intraoperative blood loss (42 ± 8.37). None of the complications reported by others have appeared in our research so far (Table 3). The results of our research further confirmed that using the TINAVI robot has many advantages. It has been reported that bone cement is a polymer that can effectively reinforce lesions, prevent pathological fractures, and relieve pain (Tschirhart et al., 2005). Bone cement can also suppress tumors via thermal damage. When it works, the average temperature of bone cement polymerization was 68°C, the maximum in 90°C, and the duration was approximately about 10 min, which could kill tumor cells and cause an embolization effect. Bone cement was disseminated into tumor blood vessels and solidified, blocking tumor blood vessels. The bone cement surrounded the lesion and blocked the further invasion of the lesion (Pusceddu et al., 2022). Zhang et al. (2020) proposed that augmented cementoplasty has several advantages, including the minimization of surgical morbidity, shorter hospital stays, and lower healthcare costs. However, the injection of bone cement has shortcomings, such as a low inactivation range of tumors, an unsatisfactory bone cement distribution, and a high leakage rate, which suggests that cement should be injected more precisely. Fortunately, with the help of an orthopedic surgical robot, we can avoid these shortcomings as much as possible. However, there are some limitations in our present study. Our study was a non-randomized controlled retrospective study with small sample size, and multicenter long-term follow-up is needed for verification. Robot preparation requires a team of engineers, and using robots has a learning curve and still needs to be popularized to benefit patients. If the infection, implant loosening, and hip dislocation complications occur during the follow-up, then THA treatment will be considered.

**TABLE 3:** The comparison of different surgical methods for acetabular metastatic carcinoma.

Year	Author	Surgical method	Surgical time (mins)	Intraoperative bleeding (mL)	Complications (e.g., infection, implant loosening, hip dislocation)
2020	Yang et al. (2020)	Tripod	137 ± 39	No blood transfusion	30%
2020	Lavignac et al. (2020)	Total Hip Arthroplasty	—	—	30%
2020	Houdek et al. (2020)	Harrington Reconstruction	318 ± 81	—	55%
2022	Plaud et al. (2022)	Harrington Procedure	135 ± 29	1,433 ± 1,177	57%
2023	Current study	TAINVI robot + Tripod + Bone cement	45 ± 10	42 ± 8.37	0% (Until now)

## 7 Conclusion

In summary, our study explored a novel minimally invasive surgical technique for treating acetabular metastasis and found that the surgical robot-assisted tripod percutaneous reconstruction technique combined with the bone cement filling technique may be a more suitable treatment in acetabular metastasis patients. This novel surgical technique may expand the indications of the tripod percutaneous reconstruction technique as a simple and easy-to-operate technique that is accurate and has the advantage of being fast. Therefore, the technology may be suitable for more widespread use in acetabular metastasis patients, but the long-term effects still need close follow-up.

## Data Availability

The raw data supporting the conclusion of this article will be made available by the authors, without undue reservation.
